# Anti-glycation properties of *Illicium verum* Hook. f. fruit *in-vitro* and in a diabetic rat model

**DOI:** 10.1186/s12906-022-03550-z

**Published:** 2022-03-19

**Authors:** Hafiz Nadeem Khan, Saima Rasheed, M. Iqbal Choudhary, Nessar Ahmed, Abdu Adem

**Affiliations:** 1 H. E. J. Research Institute of Chemistry, International Center for Chemical and Biological Sciences, University of Karachi, Karachi, 75270 Pakistan; 2 Dr. Panjwani Center of Molecular Medicine and Drug Research, International Center for Chemical and Biological Sciences, University of Karachi, Karachi, 75270 Pakistan; 3 Department of Pharmacology and Therapeutics, United Arab Emirates University, Al Ain, UAE; 4 Department of Life Sciences, Manchester Metropolitan University, Manchester, United Kingdom; 5 Department of Biochemistry, Faculty of Sciences, King Abdulaziz University, Jeddah, Saudi Arabia

**Keywords:** Diabetes, anti-glycation, *Illicium verum*, star anise, renal AGEs

## Abstract

**Background:**

Chronic hyperglycemic triggers the non-enzymatic glycation of biomolecules, resulting in the production of advanced glycation endproducts, that lead to several micro- and macrovascular complications. Therefore, the discovery of new, effective, and safe anti-glycation agents is an important need. One of the best choices for the management of diabetes is to use complementary and alternative medicinal therapies. Therefore, the present study was designed to evaluate the anti-glycation activity of ethanolic extract of *Illicium verum* Hook. f. (Star anise, a frequently used spice and medicinally important herb).

**Methods:**

The anti-glycation activity of ethanolic extract of *Illicium verum* Hook. f. was determined by using both *in-vitro* and *in-vivo* assays. HSA-fructose glycation model was employed to assess the *in-vitro* inhibition of protein glycation, additionally cross-linked AGEs (formed by incubating lysozyme with fructose) were assessed by SDS polyacrylamide gel electrophoresis. Dual inhibitory mechanisms, *i.e.*, antioxidant and metal chelating activities, were also evaluated by using DPPH, ABTS, and Fe (II)-chelation assays. Acute toxicity of *I. verum* extract was also performed (by administrating different doses *i.e.* 2,000, 1,500, 1,000, and 500 mg/kg of body weight). Finally, *in-vivo* anti-glycation potential was evaluated by 7 weeks of administration of *I. verum* extract in streptozotocin-induced diabetic rats.

**Results:**

In HSA-fructose glycation model, extract of *I. verum* showed a good inhibitory activity with IC_50_ value of 0.11±0.001 mg/mL, as compared to the standard inhibitor, rutin (IC_50_ = 0.02±0.01 mg/mL). Extract of *I. verum* showed inhibitory activity in DPPH, and ABTS radical scavenging assays with IC_50_ values of 130±1.0, and 57±2.0 μg/mL, respectively, while it was found to be inactive in the Fe^+2^-chelation assay. The extract was found to be non-toxic, and reduce the elevated blood glucose, urea, lipid, liver function parameters, and renal AGEs levels in streptozotocin-induced diabetic rats.

**Conclusions:**

These results suggest that *I. verum* supplementation might help to reduce the burden of AGEs, and may have potential in preventing diabetes-associated complications.

**Supplementary Information:**

The online version contains supplementary material available at 10.1186/s12906-022-03550-z.

## Background

Diabetes is an endocrine, and metabolic disorder. It is mainly of two types, *i.e.*, Type-Ӏ and Type-ӀӀ. Type-Ӏ diabetes mainly arises due to pancreatic β-cells destruction, while Type-ӀӀ is due to insulin resistance in which body cells do not respond properly to insulin. According to the data published by International Diabetes Federation (IDF), currently, there are around 463 million diabetic patients worldwide. Unfortunately, this will increase to 700 million in 2045 if the present situation prevails. In Pakistan, there are over 19.4 million cases of diabetes with 19.9% overall prevalence. In the United Arab Emirates, it is estimated that 15.4% of the total population is suffering from diabetes [1].

In diabetes, uncontrolled hyperglycemia results in the non-enzymatic glycation of many biomolecules (*e.g.* proteins, lipids), which ultimately leads to late diabetic complications. Glycation is a non-enzymatic reaction between bio-molecules and reducing sugars, and results in the formation of Advanced Glycation Endproducts (AGEs). Protein glycation is not only a marker of hyperglycemia but a core reason for diabetic complications. Accumulation of tissue AGEs are associated with many complications, such as nephropathy, retinopathy, neuropathy, *etc*. Therefore, there is an urgent need to develop effective, and safe inhibitors of AGEs to reduce the burden of late diabetic complications [2].

Advancements in science and technology have contributed greatly to the drug development. However, traditional medicines, such as traditional Chinese medicine, Ayurveda, and Unani medicines are of great importance, as they still been practiced in many areas of the world, and have blossomed into orderly-regulated systems of medicine. Traditional medicines have their incomparable advantages, such as abundant clinical experiences, and their unique diversity of chemical constitutes and biological activities. *Illicium verum* Hook. f. belongs to the Schisandraceae family. It is commonly used as a spice in foods and beverages. This plant is mainly cultivated in China, Vietnam, and India [3]. *Illicium verum* is an important herb in Asia, and as well as in the traditional Chinese medicine. Oil of *Illicium verum* is extracted from the pericarps of its fruit that possesses different biological activities, such as antifungal, antioxidant, and antibacterial properties [4–6]. Idm’hand and coworkers reported *I. verum* as one of the Moroccan plants with antidiabetic potentials. They reported different traditional antidiabetic remedies (decoction and maceration) from different parts of medicinal plants including *Illicium verum* [7]. Shah *et al.* reported some traditionally used hypoglycemic plants from South-East Rajasthan, India, based on data gathered from local people and their medicine men and women. They found that *Illicium verum* could also play an important role in the management of diabetes [8]. The extracts of stems and leaves of *Illicium verum* were found to inhibit the AGEs formation *in vitro* [9]. *Illicium verum* is one of the many species that contain bioactive compounds as well as a number of phenolic and flavonoid compounds, having antioxidant, and antimicrobial properties. *Illicium verum* essential oil contains anethole which has shown hypoglycemic, antioxidant, antimicrobial, hypolipidemic, and oestrogenic properties [10]. The antidiabetic potential of *Illicium verum* seeds extract was also reported through different model systems, *i.e*., α- amylase, α-glucosidase enzymes inhibition, and Glucose uptake by Yeast cells [11].

As per literature survey, there is no report of *in vivo* anti-glycation activity of *I. verum*. Therefore, our study aimed to evaluate the antidiabetic and anti-glycation activities of the dry fruit extract of *I. verum* by using a battery of *in-vitro* assays, and an *in-vivo* Streptozocin-induced diabetic model.

## Methods

### Chemicals

Human serum albumin (HSA) was purchased from Akron Biotech, USA. Lysozyme was obtained from Bio Basic Inc., Canada. Rutin, 2,2′-azino-*bis* (3-ethylbenzothiazoline-6-sulfonic acid (ABTS), 1,1-Diphenyl-2-picrylhydrazyl (DPPH), gallic acid, ascorbic acid, fructose, sodium phosphate monobasic, sodium phosphate dibasic, ferrozine, iron (II) chloride, ethylenediaminetetraacetic acid (EDTA), folin-Ciocalteu phenol reagent, aluminium chloride, sodium acetate, glycerol, glacial acetic acid, methanol, ethanol, streptozocin (STZ), and sodium azide were acquired from Sigma Aldrich, USA. Tetramethylethylenediamine (TEMED), sodium dodecyl sulfate (SDS), bromophenol blue, ammonium persulfate, acrylamide solution, Coomassie brilliant blue, glycine, and *β*-mercaptoethanol were acquired from Bio-Rad Laboratories, USA. Tris (hydroxymethyl)-aminomethane was purchased from Scharlau, Spain, while molecular markers were obtained from Thermo Scientific, USA.

### Collection of *Illicium verum* dry fruits, and preparation of extract

The present study complies with the international, national and/or institutional guidelines for the use of different parts of the plant. *Illicium verum* hook. f. dry fruits (imported from China) were taxonomically identified by Prof. Shijie Zhu. A voucher specimen (513433LY0392) is preserved for the herbarium at the National Resource Center for Chinese Materia Medica, China Academy of Chinese Medical Sciences, Beijing, China.

The fruit was thoroughly washed with distilled water and air-dried at room temperature. The dried fruits were then soaked three times in 80% ethanol for 3 days at room temperature with constant swirling after 2-3 hours. Solvent was then concentrated by rotary evaporation to obtain the crude ethanolic extract. The crude extract was then freeze-dried, and used for further studies.

### *In- vitro* glycation of human serum albumin (HSA) with fructose

Reaction was performed in 96-well plates, and each well contained 200 *μ*L reaction mixture. Briefly, human serum albumin (10 mg/mL; 50 *μ*L) was incubated with fructose (0.5 M; 50 *μ*L) in sodium phosphate buffer (pH 7.4; 80 *μ*L) both in the presence, and absence of test samples (2 mg/mL to 0.01 mg/mL; 20 *μ*L) at 37 °C for one week under aseptic conditions. Control samples without test samples were incubated under the same conditions. After seven days, the change in reaction was monitored by recording the fluorescence at 330 and 440 nm, excitation, and emission, respectively, by using a microtiter plate reader (SpectraMax M5, Molecular Devices, USA)] [12]. Percent inhibition was calculated by using the following formula:

$$\%\mathrm{Inhibition}=\left(1-\mathrm{Fluorescence}\ \mathrm{of}\ \mathrm{test}\ \mathrm{samples}/\mathrm{Fluorescence}\ \mathrm{of}\ \mathrm{the}\ \mathrm{control}\right)\ \mathrm{x}\ 100$$

### Analysis of cross-linked AGEs by SDS-PAGE

Cross-linked AGEs (formed by incubating 10 mg/mL lysozyme with 500 mM fructose for 7 days) were assessed by SDS polyacrylamide gel electrophoresis by using 15 % gels. Protein samples were prepared by mixing 10 *μ*L of sample diluting buffer with 6 *μ*L phosphate buffer (pH 7.4), and 4 *μ*L glycated lysozyme (10 mg/mL). The gels were run at 75 volts for 45 minutes initially, and then 150 volts for 70 minutes, followed by staining with Coomassie blue, and finally destained for further analysis and imaging [13].

### DPPH free radical scavenging assay

DPPH radical scavenging activity of *I. verum* ethanolic extract was measured according to the method reported by Lee and coworkers [14]. Briefly, 95 *μ*L of DPPH solution (0.3 mM, dissolved in ethanol), and 5 *μ*L of plant extract solutions (0.5 - 0.01 mg/mL in DMSO) were added in each well of a 96-well plate, and the plate was then incubated in the dark for 30 minutes at 37 °C. The plate was covered with parafilm during incubation to avoid solvent evaporation. Progress of the reaction was monitored by recording change in absorbance at 517 nm by using a microplate reader (Multiskan Go, Thermo Scientific, USA) (Lee *et al.,* 1998). Percentage of DPPH radical scavenging activity (% RSA) was calculated by using the following formula:

$$\% RSA=100-\left(\frac{\Delta {A}_{Sample}}{\Delta {A}_{Control}}\right)\times 100$$

Where RSA = radical scavenging activity and ΔA = change in absorbance.

### ABTS^·+^ antioxidant assay

Briefly, ABTS (7 mM) and 2.45 mM ammonium persulfate stock solutions were prepared in deionized water. The ABTS radical cation was generated by mixing the two solutions in 1:1 ratio and allowing them to react for 14 hours at 37 °C in the dark. The resulting solution was then diluted with ethanol to obtain an absorbance of 0.7 at 734 nm. 190 *μ*L of this diluted solution was then reacted with 10 *μ*L of plant extract solutions (0.5 - 0.001 mg/mL) and incubated at 37 °C for 7 minutes. Change in absorbance was recorded at 734 nm using the microplate-reader [15]. ABTS^**·+**^ scavenging activity was calculated by using the following formula:

$$\% RSA=100-\left(\frac{\Delta {A}_{Sample}}{\Delta {A}_{Control}}\right)\times 100$$

### Fe (II) chelation assay

The Fe^+2^ chelating ability of *I. verum* was determined in a 96-well plate in triplicate. Briefly, in each well 5 *μ*L of plant extract solutions (0.5 mg/mL; in DMSO) was reacted with 35 *μ*L of FeCl_2_ (0.3 mM; dissolved in D.I. water). The 96-well plate was incubated at 37 °C for 5 minutes, and absorbance was taken at 562 nm. In the next step, 60 *μ*L of ferrozine (0.5 mM; dissolved in D.I. water) was then added to each well, and final absorbance was recorded at 562 nm by using the microplate-reader [16, 17]. EDTA was used as the standard compound. The chelating rate was calculated by using the following formula:

$$Chelating\ Rate\ \left(\%\right)=\left[1-\frac{\left( absorbance\ of\ the\ Sample\right)}{\left( absorbance\ of\ Control\right)}\right]\times 100$$

### Estimation of total phenolics, flavonoids, and flavonols in *Illicium verum* ethanolic extracts

The total phenolic content in *I. verum* ethanolic extract was estimated according to an established method [18]. Briefly, 10 *μ*L of extract (10 g/L) was mixed with 50 *μ*L of Folin-Ciocalteu Phenol reagent (2.0 M was diluted ten-fold), and 40 μL of Na_2_CO_3_ solution (75 g/L). The reaction mixture was then incubated for 30 minutes at 20 °C, and absorbance was recorded at 765 nm by using the microplate reader. Calibration curve for gallic acid (standard) was prepared by mixing 10 μL aliquots of 0.024-0.3 mg/mL with the same reagents as described above. Results were expressed as “mg of gallic acid equivalent per gram of extract”.

The total flavonoid content was estimated according to the method reported by Brighente *et al.* [19]. Briefly, 2 % of aluminium chloride (50 *μ*L) in ethanol was mixed with 50 *μ*L of different concentrations of *I. verum* ethanolic extract (0.1 - 1.0 mg/mL). The solution was then subjected to one-hour incubation, and change in absorbance was recorded at 415 nm (methanol was used as blank) by using a microplate reader (Multiskan Go, Thermo Scientific, USA). Calibration curve for rutin as standard was prepared by using different concentrations of rutin (*i.e*., 0.5-0.025 mg/mL). Results were expressed as “mg of rutin equivalent per gram of extract”.

The total flavonol content was estimated according to colorimetric method reported by Miliauskas and coworkers [18]. Briefly, 20 *μ*L of *I. verum* extract (10 g/L) was mixed with 20 g/L AlCl_3_ (20 *μ*L; dissolved in ethanol) and 50 g/L of sodium acetate (60 *μ*L). Reaction mixture was then incubated for 2.5 hours at 20 °C, and change in absorbance was recorded at 440 nm by using the microplate reader. Different concentrations of rutin (*i.e.,* 0.5-0.025 mg/mL) were used for calibration curve. Results were expressed as mg of rutin equivalent per gram of extract. Total phenolics, flavonoids, and flavonols in the *I. verum* ethanolic extract were measured by following formula:

$$C=c.\frac{V}{m^{\prime }}$$

Where C= total phenolic, flavonoid, and flavonol content (mg/g of plant extract, in GAE, and RE, respectively); c = concentration of gallic acid established from the calibration curve (mg/mL); V = volume of the extract (mL); while, m = weight of the plant extract (g).

### GC-MS analysis

The GC–MS analysis of *Illicium verum* extract was carried out by using Agilent Technologies GC systems with GC-7890A/MS-5975C model (Agilent Technologies, Santa Clara, CA, USA) equipped with OPTIMA-5 column. Helium was used as a carrier gas with a flow rate of 1.0 ml/min. The temperature was set at 50 °C for 3 minutes with an increasing rate of 10 °C/min for 20 minutes and a holding time of about 20 min. Finally, the temperature was increased to 300 °C at 10 °C/min and was kept for 25 minutes. The mass spectrometer was operated in EI mode (70 eV). The extract was dissolved in ethanol, filtered, and then injected in a split mode [20]. The compounds were identified by comparison of their mass spectra with standards available in the NIST mass spectral library attached to the GC-MS instrument and the results obtained have been presented in Table 3.

### LC/MS characterization

LC-ESI-MS analysis (low resolution) was conducted on Bruker Amazon Speed Ion-Trap LC/MS coupled with Thermo Ultimate 3000 UPLC equipped with Agilent-C18 column of 1.8 μm, 2.1 × 50 mm. Gradient elution was performed with water/ 0.1% formic acid (solvent A) and acetonitrile (solvent B) at a constant flow rate of 0.3 ml/min. MS parameters for mass fragmentation were optimized by previous methods [21]. Results obtained have been presented in the supplementary informations (Fig. S2; Table S1).

### Ethics statement

The use and care of animals complied with the guidelines of the “Animal Care and Use Committee” of the International Center for Chemical and Biological Sciences (ICCBS), University of Karachi. Experimental procedures were approved by the “Animal Care and Use Committee” of the ICCBS, University of Karachi, Karachi-75270, Pakistan (ASP # 2018- 0022). The study has been described in accordance with the ARRIVE guidelines (Animals in research: Reporting *in vivo* experiment) [22].

### *In-vivo* determination of acute toxicity of *I. verum* extract

To determine acute toxicity, the protocol reported by Hafizur and co-workers was followed with slight modifications [23]. Sprague Dawley (SD) rats (male) were divided into 5 groups (*n*=6). Groups 1 was served as the control group, and received saline only, while animals in group 2, 3, 4, and 5 were given *I. verum* extract at doses of 500, 1,000, 1,500, and 2,000 mg/kg body weight, respectively. Each rat was given a single dose after the acclimatization period. Different doses of extracts were dissolved in water, and given *via* oral route. All animals were observed continuously for the initial four hours, and intermittently for the next six hours, followed by further monitoring for 14 hours for signs of any toxicity/lethality. After 24 hours, all animals were anesthetized with Ketamine–Xylazine cocktail (90 mg/kg, and 10 mg/Kg of body weight, respectively *via* single IP injection), and dissected for blood collection from the heart. Collected blood samples were used to measure biochemical parameters such as glucose, urea, creatinine, serum glutamic pyruvic transaminase (SGPT), Alkaline phosphatase (ALP), liver function test (LFT), *etc*.

### *In-vivo* glycation studies in STZ-induced diabetic rat model

#### Induction of diabetes in rats

Male Sprague Dawley rats (200-250 g) were obtained from *in-house* Animal Research Facility (ARF), ICCBS, University of Karachi, and acclimatized for 4 days. All animals were housed in clean cages with free access to food and water. A single dose of STZ (45 mg/kg) in citrate buffer (1 mM, pH = 4.5) was administered intraperitoneally to all rats except animals of group 1 (*i.e.* non-diabetic control). After 3 days of STZ administration, the blood glucose levels were monitored by using a glucometer (ACCU-CHEK, Performa, Roche, Switzerland). Animals having blood glucose levels above 200 mg/dL were considered as diabetic, and further re-grouped for anti-glycation studies.

#### Treatment protocol

The diabetic rats were divided into the five following groups (*n* = 6/group):

Group 1: Non-diabetic control rats

Group 2: Diabetic rats

Group 3: Diabetic rats treated with metformin (100 mg/kg)

Group 4: Diabetic rats treated with *I. verum* extracts (1000 mg/kg body weight)

Group 5: Diabetic rats treated with *I. verum* extracts (500 mg/kg body weight)

All groups received either saline or metformin or *I. verum* extract for 7 weeks. After 7 weeks, rats were fasted overnight, then sacrificed, and blood samples were collected for biochemical analysis. The biochemical parameters, such as glucose, creatinine, urea, lipid profile and liver enzymes, were analyzed through *in-house* diagnostic lab facility of the Dr. Panjwani Center for Molecular Medicine and Drug Research, ICCBS, University of Karachi, while renal AGEs were determined by using the protocol described below.

#### Estimation of AGEs level in kidneys

In order to determine the renal AGEs level, minced kidney tissues were first dilapidated with chloroform and methanol (2:1, v/v) overnight. After washing with D.I. water, the tissues were homogenized in sodium hydroxide (0.1 N), and centrifugation was carried out at 8,000×g for 15 minutes at 4 °C. The amount of AGEs was finally determined by recording the fluorescence at 370 and 440 nm excitation and emission, respectively. BSA solution (1 mg/mL in 0.1 N NaOH) was used as a reference, and its fluorescence intensity was defined as one unit of fluorescence. The fluorescence values of samples were expressed as arbitrary units (AU)/ mg protein [24].

### Statistical analysis

Microsoft Excel and GraphPad Prism 8 (GraphPad Software, San Diego, CA, USA) were used for statistical analysis and plotting graphs. SoftMaxPro 4.8 was used to analyze the results of *in vitro* experiments, which are represented as mean ± SEM from three experiments. IC_50_ values were calculated *via* EZ-FIT, Enzyme kinetics software by Perrella Scientific, Inc., USA. All results of *in vivo* experiments are expressed as mean ± SD. One-way ANOVA, followed by post hoc Dunnett’s test, was used to compare the differences among the groups. All *p*-values less than 0.05 (*p* < 0.05) were considered as statistically significant.

## Results

The present study was aimed to evaluate the anti-glycation potential of *I. verum*, a well-known spice and medicinal plant by using *in-vitro* and *in-vivo* biochemical assays. Results are described as under:

### *In-Vitro* activities

#### *In-Vitro* anti-glycation activity against HSA-fructose glycation model

To determine the inhibitory effect of the ethanolic extract of *Illicium verum* Hook. f. against protein glycation, human serum albumin-fructose glycation assay was employed. After co-incubation with HSA and fructose for 7 days (at 37 °C), the ethanolic extract of *I. Verum* exhibited good anti-glycation activity with IC_50_ = 0.11±0.001 mg/mL, when compared with the standard inhibitor, rutin (IC_50_ = 0.02±0.01 mg/mL).

#### Effect of *I. verum* extract on inhibition of lysozyme-fructose derived AGEs cross-linking

**Fig. 1:**
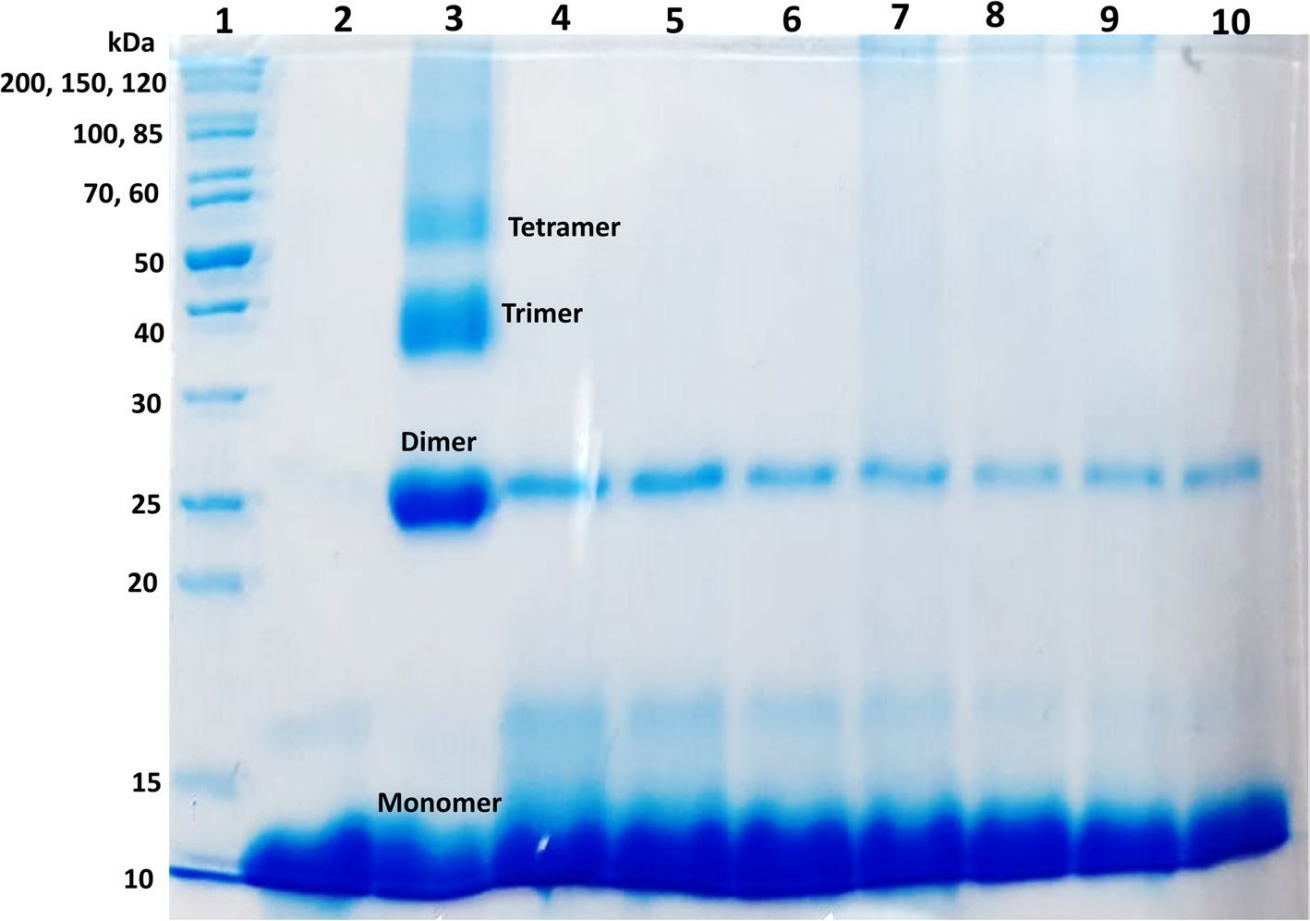
Anti-glycation activity of *Illicium verum* Hook. f. against glycated lysozyme (glycated in the presence of fructose). (1) molecular weight markers; (2) non-glycated lysozyme; (3) glycated lysozyme; (4-10): The inhibition of production of fructose-lysozyme AGEs by different concentrations of illicium verum extract. (4) 15 mg/mL, (5) 10 mg/mL, (6) 5 mg/mL, (7) 2.5 mg/mL (8) 2 mg/mL, (9) 1 mg/mL, and (10) 0.5 mg/mL. (Note: Gel is presented after cropping to improves the clarity and conciseness of the presentation. Uncropped gel can be observed in Supplementary material as Fig. S1)

Cross-linked AGEs were assessed by SDS polyacrylamide gel electrophoresis. Figure 1 shows the effect of different concentrations of ethanolic extract of *I. verum* on lysozyme-fructose-derived AGEs cross-link formation.

Glycated lysozyme (Fig. 1, lane 3) was served as the positive control, while non-glycated lysozyme (Fig. 1, lane 2) was used as a negative control to evaluate the extent of glycation/ cross-link formation. The inhibition of crosslinked AGEs by *I. verum* was performed at 15 mg/mL (Fig. 1, lane 4), 10 mg/mL (Fig. 1, lane 5), 5 mg/mL (Fig. 1, lane 6), 2.5 mg/mL (Fig. 1, lane 7), 2 mg/mL (Fig. 1, lane 8), 1 mg/mL (Fig. 1, lane 9), and 0.5 mg/mL concentrations (Fig. 1, lane 10). Glycated lysozyme showed dimer, trimer, and tetramer bands on SDS-PAGE. While in the presence of *I. verum* extract this cross-linking formation was found to be inhibited.

#### *In-vitro* antioxidant, and iron (II) chelating activities

Ethanolic extract of dry fruits of *I. verum* was also evaluated for their antioxidant activities by using *in-vitro* DPPH, and ABTS˙^+^ radical scavenging assays. Against DPPH radical-scavenging assay it showed a weak activity with an IC_50_ = 157±2.0 *μ*g/mL, when compared with reference compound, i.e., gallic acid (IC_50_ = 2.6±1.0 μg/mL). In the second step, the antioxidant capacity of *I. verum* extract was used to scavenge ABTS˙ cation, results reveal that the extract of *I. verum* scavenged the ABTS˙ cation effectively with IC_50_ = 57.4±2.0 μg/mL (Table 1). Additionally, we also evaluated *I. verum* extract for iron-chelating ability, but it was found to be inactive at 0.5 mg/mL concentration (Table 1).

**Table 1:** *In-vitro* antioxidant activities of *I. verum* ethanolic extract

	DPPH radical scavenging activity		ABTS^**.+**^ scavenging activity		Fe^**+2**^ chelation activity	
	% Inhibition	IC_**50**_ ± SEM^**a**^ (μg/mL)	% Inhibition	IC_**50**_ ± SEM^**a**^ (μg/mL)	% Chelation	IC_**50**_ ± SEM^**1**^ (μg/mL)
*I. verum*	86.6	130±1.0	99.6	57.4±2.0	7.4	NA^2^
Gallic acid^c^	97.3	2.6±1.0	-	-	-	-
Ascorbic acid^c^	-	-	98.8	3.5±6.0	-	-
EDTA^c^	-	-	-	-	99.9	21±0.6

^a^IC_50_ Values are presented in mg/mL and as mean ± standard error of mean; ^b^*NA *Not Active; ^c^Standard inhibitors for antioxidant studies

#### Total phenolics, flavonoids, and flavonols in *I. verum* ethanolic extracts

Results of total phenolic, flavonoids, and total flavonols contents estimated in the crude ethanolic extract of *I. verum* are presented in Table 2. Results showed that *I. verum* ethanolic extracts exhibited 45.98 ± 1.25 mg GAE/g phenolic contents, 45.98 ± 1.25 mg QE/g flavonoid contents, and 34.93 ± 1.45 mg RE/g flavonol contents.

**Table 2:** Total amount of phenolics, flavonoids, and flavonols in *I. verum* ethanolic extracts

Total Phenolics (mg/g) Extract (in GAE) *	Total Flavonols (mg/g) Extract (in RE) *	Total Flavonoids (mg/g) Extract (in RE) *
45.98 ± 1.25	28.68 ± 0.29	34.93 ± 1.45

GAE is gallic acid equivalent, while RE is rutin equivalent. *Data are presented as mean ± SEM (*n* = 3)

#### GC-MS analysis of ethanolic extract of *Illicium verum*

Results of GC-MS analysis of the ethanolic extract of *I. verum* are presented in Table 3. The most abundant compounds identified were 1-methoxy-4-(1-propenyl)-benzene (anethole) and 4-methoxy-benzaldehyde (with 100%, and 33.36% peak area, respectively).

**Table 3:** Compounds identified in the ethanolic extracts of *I. verum* by GC-MS

S. No.	Compound	RT	Molecular Formula	Molecular Weight	Peak Area %
1.	*N*-Methoxy- Formamide	6.32	C_2_H_5_NO_2_	75	3.65
2.	Ethylbenzene	7.53	C_8_H_10_	106	4.76
3.	*p*-Xylene	7.68	C_8_H_10_	106	16.08
4.	1-Methyl-4-isopropyl-1-cyclohexen-4-ol	13.27	C_10_H_18_O	154	1.75
5.	4-Methoxyphenol	13.71	C_7_H_8_O_2_	124	2.89
6.	4-Methoxybenzaldehyde or 4-Anisaldehyde	14.43	C_8_H_8_O_2_	136	33.36
7.	Anethole	14.87	C_10_H_12_O	148	100
8.	2-Methoxy-3-(2-propenyl)- Phenol	15.85	C_10_H_12_O_2_	164	4.32
9.	*p*-Acetonylanisole	16.23	C_10_H_12_O_2_	164	28.22
10.	*N*(1)-[(2-ethoxy-3 methoxyphenyl)methyl]- 1*H*-1,2,3,4-Tetrazole-1,5-diamine	18.07	C_11_H_16_N_6_O_2_	264	13.82
11.	4-Methoxy-α-oxo-benzeneacetic acid	18.51	C_9_H_8_O_4_	180	6.21
12.	2-Hydroxy-2-(4-methoxy-phenyl)-*N*-methyl-acetamide	18.72	C_10_H_13_NO_3_	195	31.81
13.	1-(3-Methyl-2-butenoxy)-4-(1-propenyl)benzene	20.28	C_14_H_18_O	202	16.76
14.	(*E*)-9-Octadecenoic acid ethyl ester	39.51	C_20_H_38_O_2_	310	11.09
15.	3-(2,4-Dimethoxyphenyl)butan-2-one	45.22	C_12_H_16_O_3_	208	6.96
16.	4-Methoxy-6,12-(ethylideno)- dibenzo [b,f]1,5-dioxacyclooctane	46.11	C_17_H_16_O_3_	268	12.75
17.	Estragole	13.53	C_10_H_12_O	148	2.43

### *In-vivo* activities

#### Acute toxicity studies

Acute toxicity of the ethanolic fruit extract of *I. verum* was evaluated by orally administering different doses of *I. verum*, *i.e*., 500, 1,000, 1,500, and 2,000 mg/kg of body weight (in water), while the control group received only vehicle. Animals in each group were observed for mortality and behavioral changes during the next 24 hours. No death of animals was recorded, but behavioral changes (such as becoming dizzy, wobbly, or slow and sleepy) were observed with doses of 2,000 and 1,500 mg/kg, with the increase in certain biochemical parameters (Table 4). Briefly, in the group of animals treated with *I. verum* at a dose of 1,500 mg/kg of body weight, increase in different biochemical parameters were observed as compared to the control group, such as urea (35.3 ± 8.9 *vs*. 27.75 ± 0.95), and ALP (850.3 ± 11.13 *vs*. 830.2 ±23.21). In the group of animals treated with *I. verum* extract at a dose of 2,000 mg/ kg of body weight, some biochemical parameters were also found to be elevated, such as urea (38.3 ± 3.0 *vs*. 27.75 ± 0.95), total bilirubin (0.3 ± 0.02 *vs*. 0.21 ± 0.02), and ALP (858 ± 16.9 *vs*. 830.2 ±23.21) (Table 4). While *I. verum* administered at a dose of 1,000 mg/kg, and 500 mg/kg of body weight do not affect these biochemical parameters as compared with the control animals (Table 4). Therefore, based on these results, *I. verum* extract at doses of 1,000 mg/kg, and 500 mg/kg of body weight were selected for further *in-vivo* anti-glycation studies.

**Table 4:** Various biochemical parameters in normal Sprague Dawley rats after a single administration of different doses of *I. verum* extract

Groups	Glucose levels (mg/dL)	Urea (mg/dL)	Creatinine (mg/dL)	Total Bilirubin (mg/dL)	Direct Bilirubin (mg/dL)	SGPT (U/L)	ALP (U/L)	GAMMA GT (U/L)
Control	106 ± 13.0	27.75 ± 0.95	0.87 ± 0.05	0.21 ± 0.02	0.187 ± 0.01	74 ± 6.1	830.2 ±23.21	5.5 ± 0.39
*I. verum* 500 mg/ kg	96 ± 6.21	29.2 ± 6.1	0.87 ± 0.09	0.18 ± 0.05	0.180 ± 0.04	76.7 ± 21.2	829 ± 6.68	5.5 ± 0.9
*I. verum* 1,000 mg/ kg	108.6 ± 15.8	34.0 ± 1.7	0.83 ± 0.11	0.2 ± 0.02	0.170 ± 0.02	62.3 ± 15.5	815 ± 18.5	5.6 ± 0.5
*I. verum* 1,500 mg/ kg	113.3 ± 10.01	35.3 ± 8.9	0.83 ± 0.1	0.2 ± 0.06	0.180 ± 0.03	68.3 ± 11.6	850.3 ± 11.13^*^	6.0 ± 1.0
*I. verum* 2,000 mg/ kg	118.3 ± 6.65	38.3 ± 3.0	0.8 ± 00	0.3 ± 0.02	0.181 ± 0.01	55.0 ± 2.8	858 ± 16.9^*^	6.5 ± 0.7

Each value presents mean ± S.D. (*n* = 6)

500 mg/kg, 1,000 mg/kg, 1,500 mg/kg and 2,000 mg/kg refer to different doses of *Illicium verum* administered to normal rats

* Statistical significance *vs.* normal control (*p* < 0.001)

#### Anti-glycation studies in STZ-induced diabetic rat model

STZ administration at a dose of 45 mg/kg resulted in the successful development of the *in-vivo* diabetic model, resulting in significantly increased levels of various biochemical parameters (such as blood glucose, urea, creatinine, direct bilirubin) in diabetic rats when compared with non-diabetic rats.

**Change in Body weight:** As presented in Table 5, changes in the body weights of all animals in different groups were observed during this study. Body weight in the diabetic group was significantly (*p* < 0.05) decreased when compared to the rats in the non-diabetic group. Additionally, there is also a significant decrease in the body weight of the diabetic group was observed when we compare body weight at Week 1 and at week 7. We observed that body weights of rats were found to be improved in all treatment groups after second week , *i.e*., metformin-treated (week 2= 187.00 ± 10.6 g *vs*. week 7= 219.65±13.20 g), *I. verum* 1,000 mg/kg of body weight (week 2=249.00 ± 18.60 g *vs*. week 7= 276.70 ± 19.90 g), and *I. verum* 500 mg/kg of body weight (week 2= 221.60 ± 13.50 g *vs*. week 7= 289.31±17.81 g). Improvement in the body weight of *I. verum* treated groups was recorded throughout the study period, *i.e.* during 7 weeks.

**Table 5:** Change in body weight (g) of control and groups treated with *I. verum* ethanolic extract during *in-vivo* anti-glycation studies

Groups	Body weight (g)						
	Week 1	Week 2	Week 3	Week 4	Week 5	Week 6	Week 7
Non-diabetic	213.80 ± 2.16	221.00 ± 3.80	228.40 ± 5.41	235.20 ± 5.80	241.60 ± 7.50	253.60 ± 6.40	264.20 ± 6.00
Diabetic	238.50 ± 13.80	209.20 ± 11.40	201.50 ± 11.50	193.70 ± 11.50	184.20 ± 12.40	175.00 ± 14.70	162.21 ± 11.60
Metformin treated (100 mg/kg)	214.60 ± 20.10	187.00 ± 10.6	198.30 ± 17.20	202.60 ± 19.10	208.60 ± 11.80	215.00 ± 14.70	219.65 ± 13.20
*I. verum* (1000 mg/kg)	289.00± 18.40	249.00 ± 18.60	252.00 ± 15.75	255.00 ± 18.92	261.50 ± 19.70	268.20 ± 17.90	276.70 ± 19.90
*I. verum* (500 mg/kg)	241.60 ± 17.60	221.60 ± 13.50	234.60 ± 18.700	251.30 ± 16.80	270.30 ± 16.90	276.30 ± 19.00	289.31 ± 17.81

Values are expressed as mean ± S.D. (*n* = 6)

**Fig. 2:**
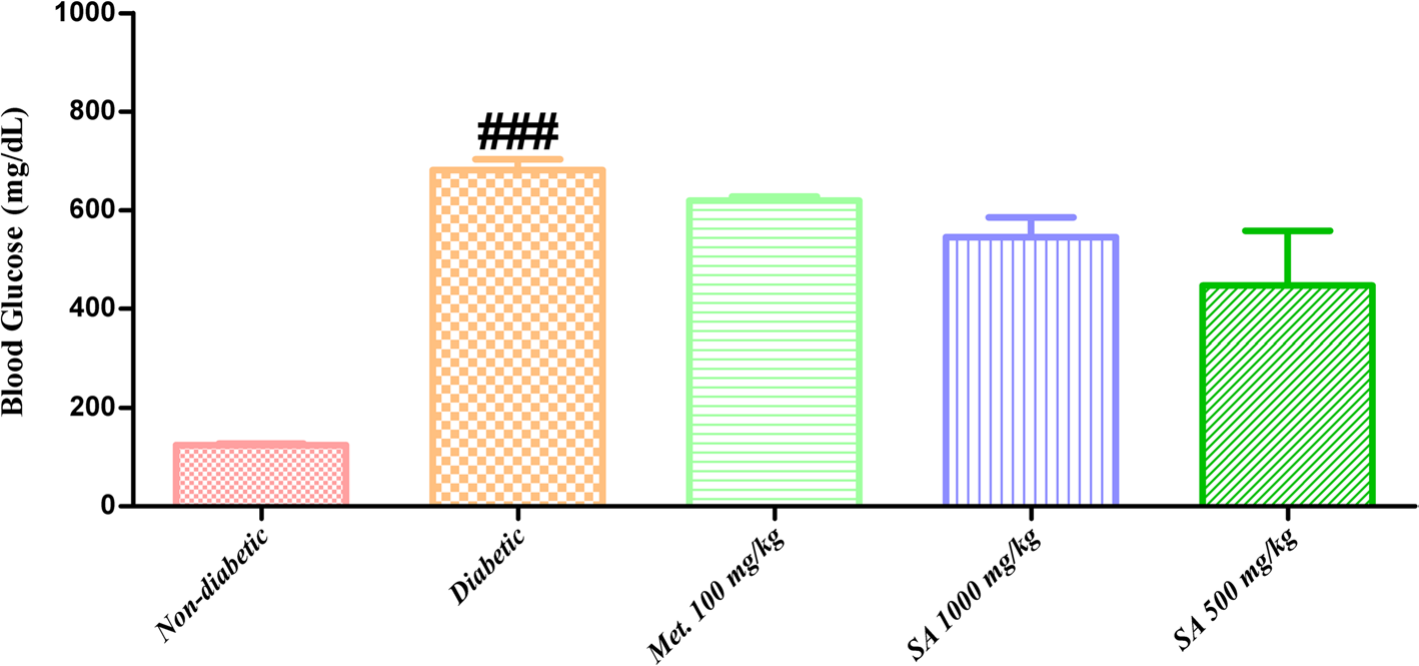
Effect of different doses of *I. verum* fruit extract (ethanolic) on blood glucose level of STZ-induced diabetic rats. During this study, diabetic rats were given different doses of dry fruit extract of *I. verum* (*i.e.,* 1,000 and 500 mg/kg of body weight; denoted as SA 1,000 mg/kg, and SA 500 mg/kg, respectively), metformin (100 mg/kg; denoted as Met. 100 mg/kg), while non-diabetic rats received normal saline only. Each value presents mean ± S.D., *n* = 6. ### Statistical significance *vs.* normal control (*p* < 0.05)

**Blood glucose levels:** Data presented in Fig. 2 reveal that diabetic rats showed a significant increase (*p* < 0.05) in blood glucose levels than the normal rats. During this study, a progressive improvement in blood glucose levels was observed in *I. verum* treated groups as compared to the diabetic group. Interestingly, *I. verum* at a dose of 500 mg/kg of body weight was found to be more effective than 1,000 mg/kg of body weight in decreasing blood glucose levels.

**Fig. 3:**
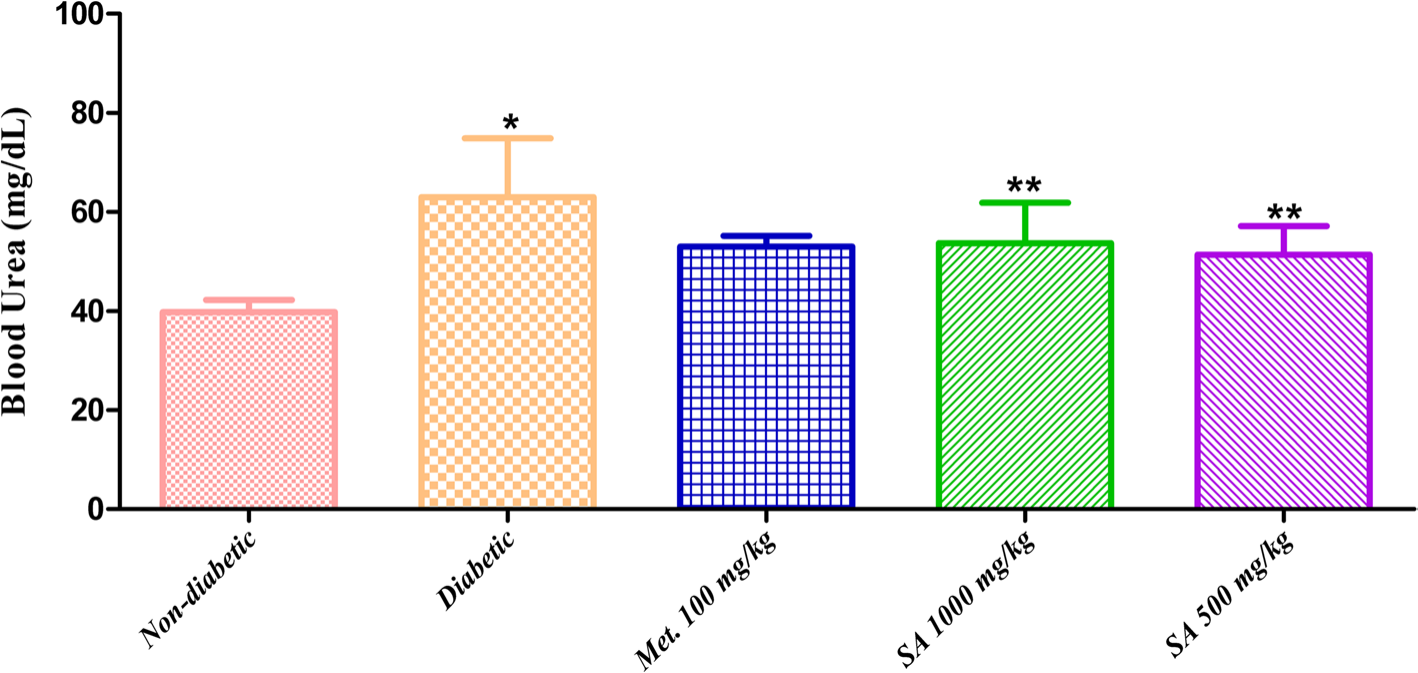
Effect of different doses of *I. verum* fruit extract (ethanolic) on blood urea levels of STZ-induced diabetic rats. During studies, diabetic rats were given different doses of dry fruit extract of *I. verum* (i.e., 1,000 and 500 mg/kg of body weight; denoted as SA 1,000 mg/kg, and SA 500 mg/kg, respectively), metformin (100 mg/kg; denoted as Met. 100 mg/kg), while non-diabetic rats received normal saline for 7 weeks. (Each value presents mean ± S.D., *n* = 6. *Statistical significance vs. normal control (*p* < 0.05), ** nonstatistical significance vs normal control (*p* >0.05)

**Fig. 4:**
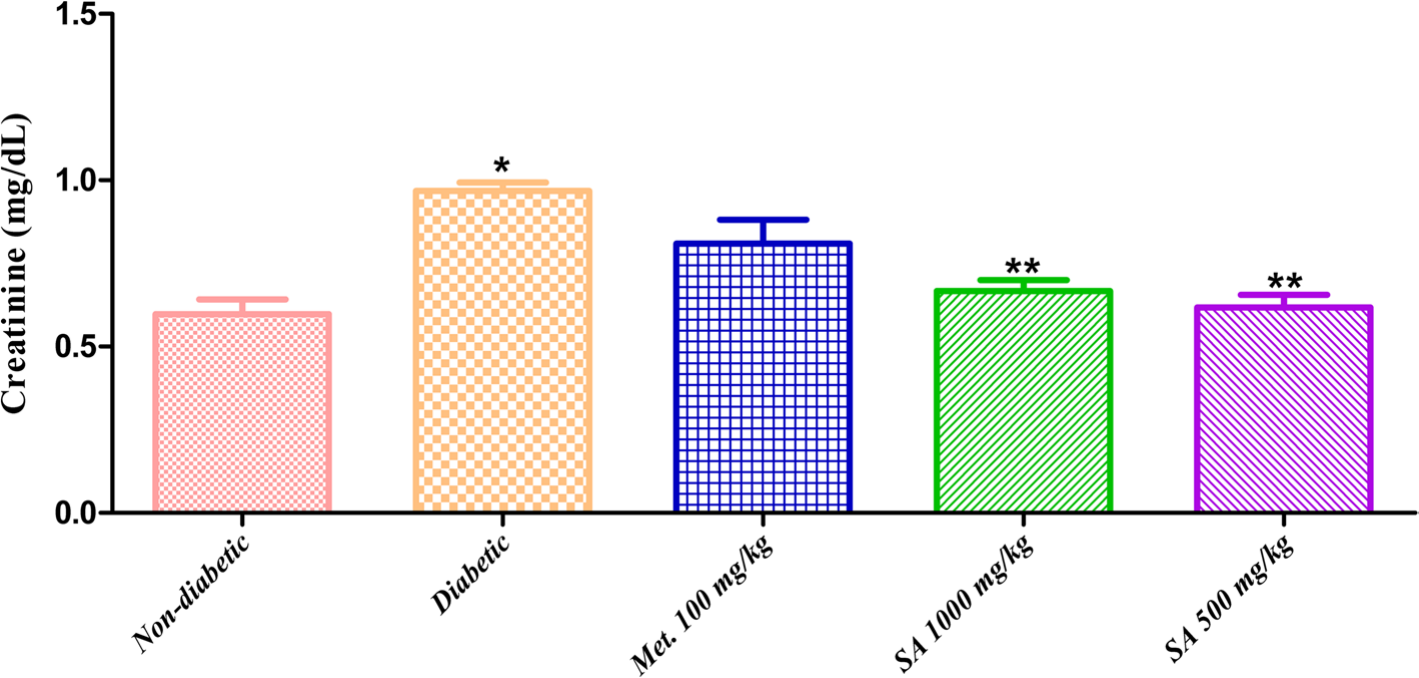
Effect of different doses of *I. verum* fruit extract (ethanolic) on serum creatinine levels of STZ-induced diabetic rats. During studies, diabetic rats were given different doses of dry fruit extract of *I. verum* (*i.e.,* 1,000 and 500 mg/kg of body weight; denoted as SA 1,000 mg/kg, and SA 500 mg/kg, respectively), Metformin (100 mg/kg; denoted as Met. 100 mg/kg), while non-diabetic rats received normal saline for 7 weeks. (Each value presents mean ± S.D., *n* = 6. *Statistical significance vs. normal control (*p* < 0.05), ** nonstatistical significance vs normal control (*p* >0.05)

**Blood urea and creatinine:** The levels of blood urea and creatinine in normal, diabetic, and *I. verum* treated groups are presented in Fig. 3 and Fig. 4. Urea and creatinine levels in the plasma were increased significantly (*p* < 0.05) in diabetic rats than in the normal/non-diabetic group. Administration of *I. verum* extract decreased the plasma urea levels non-significantly (*p* >0.05), while its administration decreased the serum creatinine levels significantly (*p* < 0.05). Unfortunately, no improvement in the glycated haemoglobin (HbA1c) levels were observed after treatment with *I. verum* at doses of 1,000 and 500 mg/kg of body weight.

**Fig. 5:**
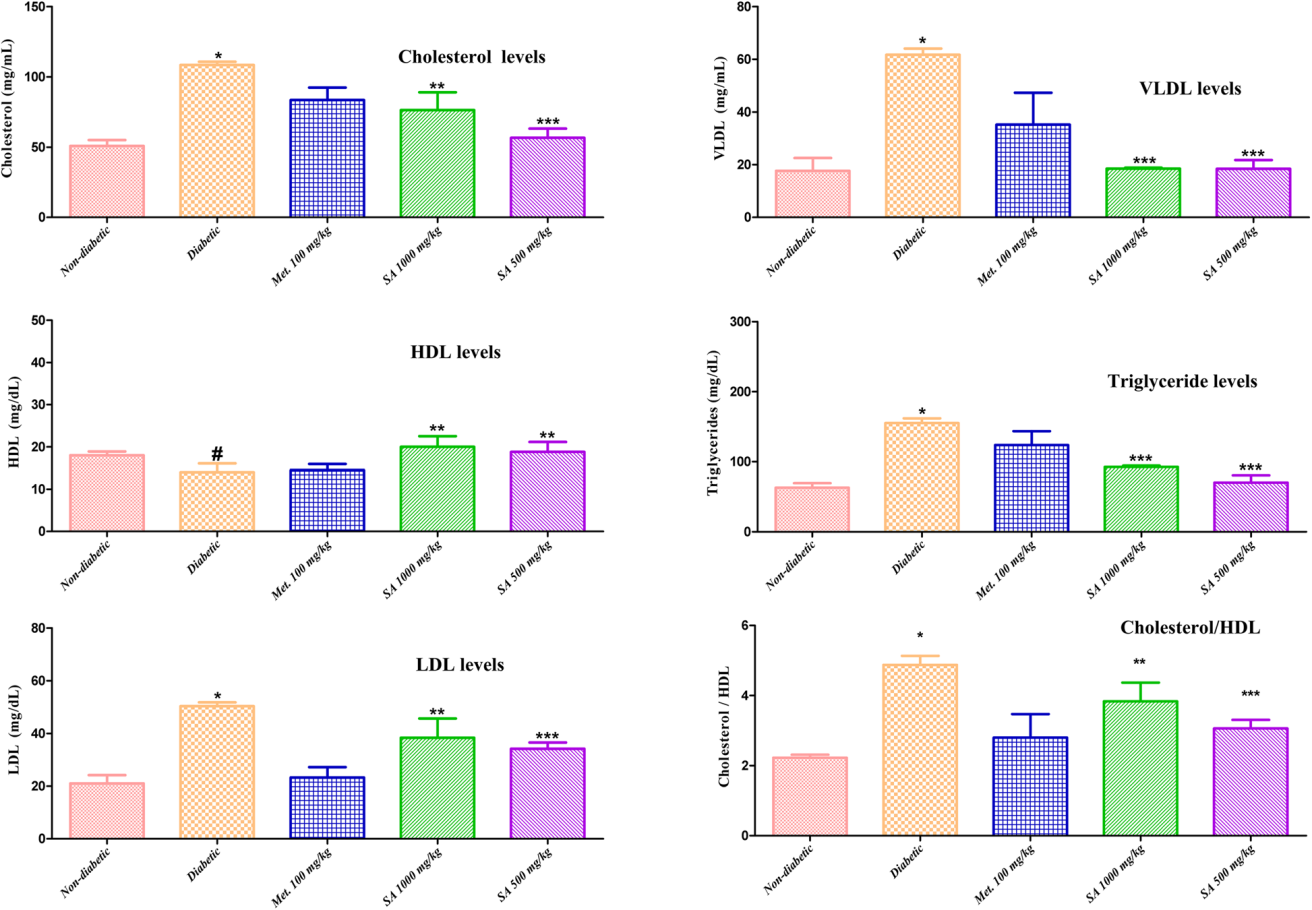
Effect of different doses of *I. verum* fruit extract (ethanolic) on serum lipid levels of STZ-induced diabetic rats. During studies, diabetic rats were given different doses of the dry fruit extract of *I. verum* (*i.e.,* 1,000 and 500 mg/kg of body weight; denoted as SA 1,000 mg/kg, and SA 500 mg/kg, respectively), metformin (100 mg/kg; denoted as Met. 100 mg/kg), while non-diabetic rats received normal saline for six weeks. (Each value presents mean ± S.D., *n* = 6. *Statistical significance vs. normal control (*p* < 0.05), # nonstatistical significance vs normal control (*p* > 0.05), ** nonstatistical significance vs diabetic group (*p* > 0.05), ***statistical significance vs. diabetic group (*p* < 0.05)

**Serum lipid levels:** Fig. 5 shows the change in the serum lipid levels (*i.e.,* cholesterol, high-density lipoprotein (HDL), low-density lipoprotein (LDL), very low-density lipoprotein (VLDL), and triglycerides levels) in normal, diabetic, and *I. verum* extract treated animals in each group. Induction of diabetes resulted in a significant increase (*p* < 0.05) in the serum cholesterol, cholesterol/HDL ratio, LDL, VLDL, and triglycerides levels than in the normal rats, and a non-significant (*p* > 0.05) decrease serum HDL levels. While treatment of rats (diabetic) with the ethanolic extract of the dry fruits of *I. verum* at a dose of 1,000 mg/kg of body weight decreased the serum cholesterol, LDL levels, cholesterol/HDL (*p* > 0.05; non-significantly), VLDL, and triglycerides levels (*p* < 0.05; significantly), as compared to the non-treated diabetic rats. Treatment of diabetic rats with ethanolic extract of the dry fruits of *I. verum* at a dose of 500 mg/kg of body weight decreased the serum cholesterol, LDL, VLDL, and triglycerides levels (*p* < 0.05; significantly), as compared to the diabetic rats. We also observed that treatment of diabetic rats with *I. verum* extract at doses of 500, and 1,000 mg/kg of body weight caused an increase in serum HDL levels when compared with diabetic group (*p* > 0.05; non-significant).

**Fig. 6:**
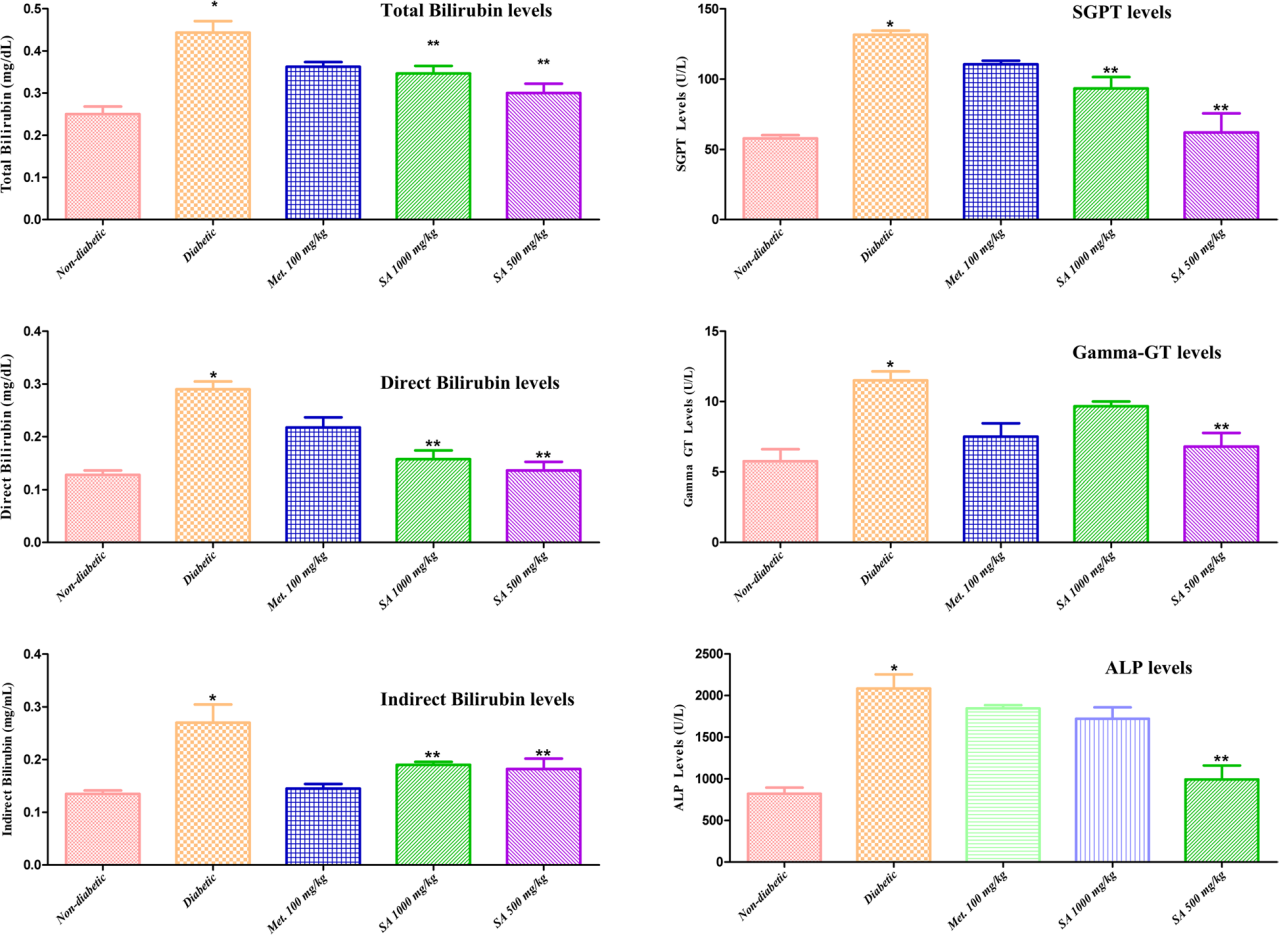
Effect of different doses of *I. verum* fruit extract (ethanolic) on liver test parameters of STZ-induced diabetic rats. During studies, diabetic rats were given different doses of the dry fruit extract of *I. verum* (*i.e.,* 1,000 and 500 mg/kg of body weight; denoted as SA 1,000 mg/kg, and SA 500 mg/kg, respectively), metformin (100 mg/kg; denoted as Met. 100 mg/kg), while non-diabetic rats received normal saline for 6 weeks. (Each value presents mean ± S.D., *n* = 6. *Statistical significance *vs.* normal control (*p* < 0.05), **statistical significance vs. diabetic group (*p* < 0.05)

**Liver test parameters:** Total bilirubin, direct bilirubin, indirect bilirubin, SGPT, Gamma-Glutamyl Transferase (Gamma-GT), and ALP levels of STZ-induced diabetic rats treated with the ethanolic extract of *I. verum* are shown in Fig. 6.

Results revealed that administration of *I. verum* at doses of 500, and 1,000 mg/kg of body weight significantly decreased (*p* < 0.05) the plasma total bilirubin, direct bilirubin, indirect bilirubin, SGPT, Gamma-GT, and ALP levels as compared to the diabetic group.

**Fig. 7:**
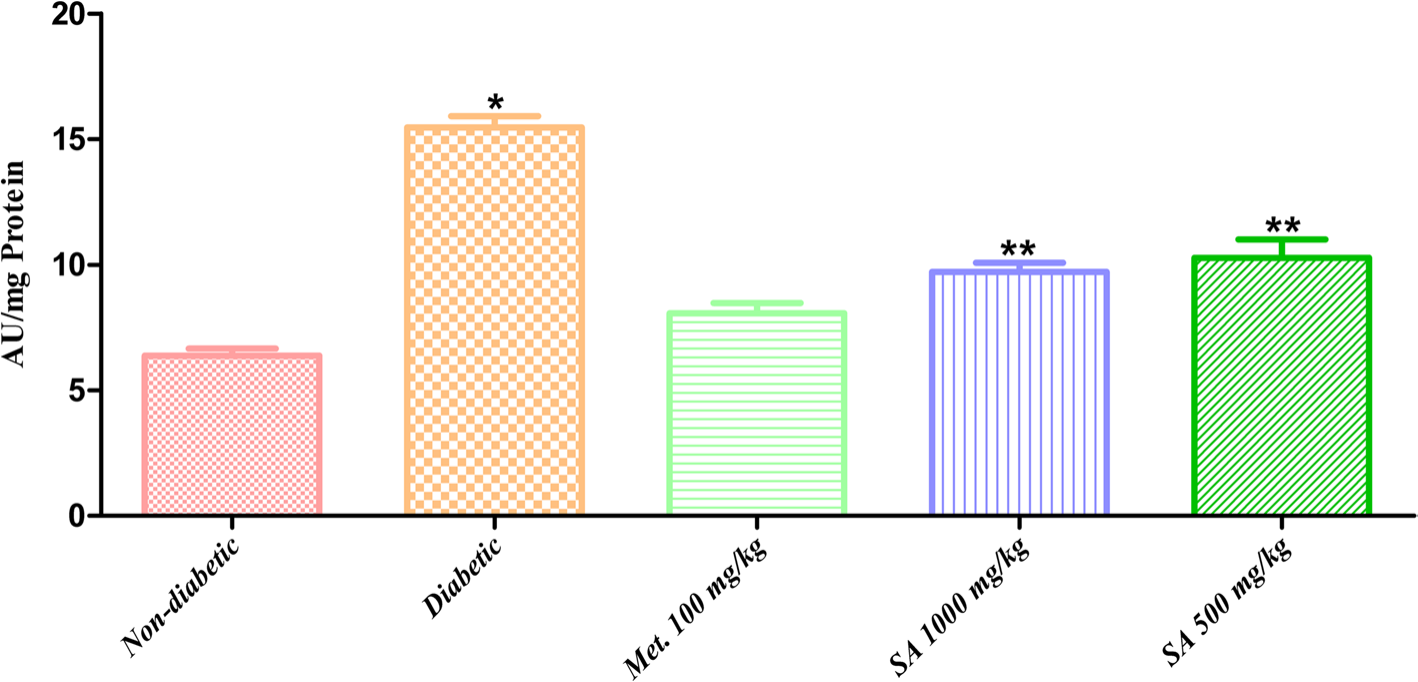
Effect of different doses of *I. verum* fruit extract on renal AGEs in diabetic rats. During studies, diabetic rats were given different doses of the dry fruit extract of *I. verum* (i.e., 1,000 and 500 mg/kg of body weight; denoted as SA 1,000 mg/kg, and SA 500 mg/kg, respectively), metformin (100 mg/kg; denoted as Met. 100 mg/kg), while non-diabetic rats received normal saline for 7 weeks (Each value presents mean ± S.D., *n* = 6. *Statistical significance *vs.* normal control (*p* < 0.05), **statistical significance vs. diabetic group (*p* < 0.05)

**Renal AGEs:** STZ-induced diabetes caused a significant increase (*p* < 0.05) in the renal AGE content of the diabetic group as compared to the normal/ non-diabetic rats (Fig. 7). renal AGEs levels were reduced significantly on treatment with the dry fruit extract of *I. verum* (1,000 and 500 mg/kg of body weight).

## Discussion

### *In-Vitro* studies

Medicinal plant-based anti-glycation and antioxidant remedies are now attracting major scientific and public interest, mainly because of their perceived gentleness. In the present work anti-glycation potential of the ethanolic extract of dry fruit of *I. verum* (a well-known spice and medicinal plant) is evaluated by using a battery of *in-vitro*, and *in-vivo* assays (by using STZ-diabetic rat model).

Results revealed that the *I. verum* extract exhibited good *In-vitro* anti-glycation activity with IC_50_ = 0.11±0.001 mg/mL, when compared with the standard inhibitor, rutin (IC_50_ = 0.02±0.01 mg/mL). In the next step, cross-linked AGEs formation was assessed by SDS polyacrylamide gel electrophoresis. Fig. 1 shows the effect of different concentrations (*i.e*., 15, 10, 5, 2.5, 2, 1, and 0.5 mg/mL of ethanolic extract of *I. verum* on lysozyme-fructose derived AGEs cross-link formation. The molecular weight of the non-glycated lysozyme is around 14.3 kDa. High molecular weight protein products were observed when lysozyme was incubated with fructose, *i.e.,* dimers (molecular weight of ~28 kDa), trimers (molecular weight of ~45 kDa), and tetramers (approximate molecular weight of 55 kDa) (Fig. 1; lane 3). These high molecular weight protein products were not observed in the non-glycated lysozyme sample (Fig. 1; lane 2). It was found that all concentrations of *I. verum* inhibited the trimer and tetramer formation (Fig 1, lanes 4-10). These results suggested that *I. verum* extract can inhibit the formation of cross-linked AGEs in the model protein system.

Anti-glycation activities of plant extracts are widely attributed to their antioxidant properties. In this regard, antioxidant properties of *I. verum* extract were evaluated by using *in-vitro* DPPH, and ABTS˙^+^ radical scavenging assays. These assays are well-known markers for determining the antioxidant capacity of any sample. Therefore, free radical scavenging capacity of the *I. verum* extract was first evaluated by using DPPH radical-scavenging assay, and a weak activity with an IC_50_ = 157±2.0 μg/mL was observed, when compared with reference compound, *i.e.*, gallic acid (IC_50_ = 2.6±1.0 μg/mL). In the second step, the antioxidant capacity of *I. verum* extract was assessed to scavenge ABTS˙ cation. Results reveal that the extract of *I. verum* showed a weak ABTS˙ cation scavenging activity (IC_50_ = 57.4±2.0 μg/mL) when compared with standard, ascorbic acid (IC_50_ = 3.5±6.0 μg/mL) (Table 1). In the next step, we also evaluated *I. verum* extract for iron-chelating ability, but it was found to be inactive (showed only 7.4% iron-chelating activity), revealing that the ethanolic extract of *I. verum* cannot chelate effectively with the iron metal at 0.5 mg/mL concentration (Table 1).

Flavonoids and flavonols are among the most important groups of natural compounds and are known to inhibit the glycation of biomolecules through scavenging free radicals or effectively chelate metal ions. It is reported that medicinal plants with high phenolic, flavonoid, and flavonol content can effectively inhibit the glycation reaction [24, 25]. As shown in Table 2, the concentration of total phenolic, total flavonoid, and total flavonols contents of *I. verum* were found to be 45.98 ± 1.25 mg gallic acid equivalent per gram (GAE/g), 45.98 ± 1.25 mg quercetin equivalent per gram (QE/g), and 34.93 ± 1.45 mg rutin equivalent per gram (RE/g), respectively.

GC-MS analysis of the ethanolic extract of *I. verum* helped us to identify 1-methoxy-4-(1-propenyl)-benzene (anethole), 4-methoxy-benzaldehyde, and 2-Hydroxy-2-(4-methoxy-phenyl)-N-methyl-acetamide (with 100%, 33.36%, and 31.81% peak area, respectively) as the most abundant compounds (Table 3). We also perform LC-MS analysis of ethanolic extract of illicium verum, and able to identify only seven compounds (kindly see Fig. S2 and Table S1 of supplementary informations). There are different secondary metabolites reported form *I. verum*, such as phenylpropanoids, phenols, organic acids, glycosides, flavonoids, neolignans, mono and sesquiterpeniods. *Trans*-Anethole is a major constituent of *I. verum* which is extensively used in food, perfume, and pharmaceutical industries [25, 26]. Sharafan *et al*., summarized different compounds reported from *I. verum*, such as phenolic compounds (*Trans*-anethole, cis-anethole, estragole, Illiverin A, *etc*.), monoterpenoids (α-Pinene, p-cymene, limonene, linalool, terpinen-4-ol, *etc*.), sesquiterpenoids (α-Phellandrene, α-muurolene, veranisatins A-C, etc.), flavonoids (chalcones, kaempferol and glucosides, quercetin and glucosides, etc.), fatty acids (Linoleic acid, stearic acid, myristic acid, etc.) [27]. *Trans*-anethole possesses antibacterial, antifungal, antioxidant, anti-inflammatory and anti-obesity properties [24]. Sheikh *et al*., reported that anethole showed reduction in the levels of glucose, and HbA1c levels. They reported that upon administration of *trans*-anethole, the altered levels of hexokinase, glucose-6-phosphate dehydrogenase, and glucose-6-phosphatase and fructose-1,6-bisphosphatase in the liver and kidney of diabetic rats significantly reverted to near normal levels [28]. Another commonly reported secondary metabolite reported from *I. verum* is Estragole, a naturally occurring phenylpropanoids. Estragole has many biological effects, including antioxidant and antimicrobial activities [29]. Therefore, based on literature and our findings it may say that the anti-glycation and antioxidant activities of *I. verum* extract are might be due to the presence of phenolic, phenylpropanoids, flavonoids, and terpenoids compounds.

### *In-Vivo* studies

*In-vitro* activities of the dry fruit extract of *I. verum* plant as an anti-glycating, and antioxidant agent, with significant phenolic, flavonoid, and flavonols content, motivated us to evaluate the potential use of this extract for the management of diabetes in STZ-induced diabetic rat model. In this regard, we first evaluated its acute toxicity in healthy rats, then its anti-glycation activity in the STZ-induced diabetic rat model was assessed.

### *In-vivo* acute toxicity studies

Acute toxicity of the ethanolic fruit extract of *I. verum* was evaluated by orally administering different doses of *I. verum*, *i.e*., 500, 1,000, 1,500, and 2,000 mg/kg of body weight (in water), while the control group received only vehicle. Animals in each group were observed for mortality and behavioral changes during the next 24 hours. No death was recorded, but behavioral changes (such as becoming dizzy, wobbly, or slow and sleepy) were observed with doses of 2,000 and 1,500 mg/kg, with the increase in certain biochemical parameters (Table 4). Briefly, in the group of animals treated with *I. verum* at a dose of 1,500 mg/kg of body weight, increase in different biochemical parameters were observed when compared with the control group, such as urea (35.3 ± 8.9 mg/dL *vs.* 27.75 ± 0.95 mg/dL), and ALP (850.3 ± 11.13U/L *vs.* 830.2 ±23.21 U/L). In the group of animals treated with *I. verum* extract at a dose of 2,000 mg/ kg of body weight, some biochemical parameters were also found to be elevated, such as urea (38.3 ± 3.0 mg/dL vs 27.75 ± 0.95 mg/dL), total bilirubin (0.3 ± 0.02 mg/dL *vs* 0.21 ± 0.02 mg/dL), and ALP (858 ± 16.9 U/L *vs.* 830.2 ±23.21 U/L) (Table 4). While *I. verum* administered at a dose of 1,000 mg/kg, and 500 mg/kg of body weight do not affect these biochemical parameters when compared with the animals in control group (Table 4). Therefore, based on these results, *I. verum* extract at doses of 1,000 mg/kg, and 500 mg/kg of body weight were selected for *in-vivo* anti-glycation studies.

### *In-vivo* anti-glycation studies

During current studies, STZ administration resulted in the successful development of diabetic rat model, with significantly increased levels of various biochemical parameters (such as blood glucose, urea, creatinine, and direct bilirubin) in diabetic rats when compared with non-diabetic rats. Administration of ethanolic extract of dry fruits of *I. verum* decreased the elevated level of blood glucose, urea, creatinine, serum lipids, and liver test parameters (*i.e.,* total bilirubin, direct bilirubin, indirect bilirubin, SGPT, Gamma GT, and ALP levels).

Body weights of all animals in different groups were recorded during the whole period *i.e*., seven weeks of this study and presented in Table 5. Results showed that body weight in the diabetic group was significantly (*p* < 0.05) decreased when compared with rats in the non-diabetic group. While body weights of rats were improved in all treatment groups, *i.e.,* metformin-treated (219.65±13.20 g), *I. verum* 1,000 mg/kg of body weight (276.70 ± 19.90 g), and *I. verum* 500 mg/kg of body weight (289.31±17.81 g) as compared to the diabetic group (162.21 ± 11.60 g). The second important parameter that was observed during this study was the change in the blood glucose levels of animals in all groups. Data revealed that diabetic rats have a significant increase (*p* < 0.05) in blood glucose levels than the normal rats. This is due to fact that streptozotocin (STZ) damages the pancreatic islet β-cells, and prevents insulin secretion, thus results in type-1 diabetes. Progressive improvement (decrease) in blood glucose levels was observed in *I. verum* treated groups as compared to the diabetic group (Fig. 2). Interestingly, *I. verum* at a dose of 500 mg/kg of body weight was found to be more effective than 1,000 mg/kg of body weight in decreasing blood glucose levels. Changes in the levels of blood urea and creatinine in normal, diabetic, and *I. verum* treated groups are presented in Fig. 3 and Fig. 4. Results showed that urea and creatinine levels in the plasma were increased significantly (*p* < 0.05) in diabetic rats than in the normal/non-diabetic group. Administration of *I. verum* extract decreased the plasma urea levels non-significantly (*p* >0.05), while its administration decreased the serum creatinine levels significantly (*p* < 0.05). *I. verum* at a dose of 500 mg/kg of body weight was again found to be more effective than 1,000 mg/kg of body weight to decrease these parameters. Unfortunately, no improvement in the HbA1c levels was observed after treatment with *I. verum* at doses of 1,000 and 500 mg/kg of body weight.

Serum lipid levels (*i.e.,* cholesterol, HDL, LDL, VLDL, and triglycerides levels) in normal, diabetic, and treated animals in each group are presented in Fig. 5. As expected, induction of diabetes resulted in a significant increase (*p* < 0.05) in the serum cholesterol, cholesterol/HDL ratio, LDL, VLDL, and triglycerides levels than in the normal rats, and a non-significant (*p* > 0.05) decrease serum HDL levels. Our findings showed that treatment of diabetic rats with the ethanolic extract of the dry fruits of *I. verum* at a dose of 1,000 mg/kg of body weight decreased the serum cholesterol, LDL levels, cholesterol/HDL (*p* > 0.05; non-significantly), VLDL, and triglycerides levels (*p* < 0.05; significantly), as compared to the non-treated diabetic rats. Treatment of diabetic rats with ethanolic extract of the dry fruits of *I. verum* at a dose of 500 mg/kg of body weight decreased the serum cholesterol, LDL, VLDL, and triglycerides levels (*p* < 0.05; significantly), as compared to the diabetic rats. Additionally, treatment of diabetic rats with *I. verum* extract at doses of 500, and 1,000 mg/kg of body weight caused an increase in serum HDL levels as compared to the diabetic group (*p* > 0.05; non-significant).

Liver test parameters, such as total bilirubin, direct bilirubin, indirect bilirubin, SGPT, Gamma GT, and ALP levels of STZ-induced diabetic rats were found to be significantly increased (*p* < 0.05) than the normal control group. We find out that administration of *I. verum* at doses of 500, and 1,000 mg/kg of body weight significantly decreased (*p* < 0.05) the plasma total bilirubin, direct bilirubin, indirect bilirubin, SGPT, Gamma GT, and ALP levels as compared to the diabetic group. Among all liver test parameters, elevated levels of ALT, and SGPT are specific indicators of liver damage. This elevation causes an increase in ketogenesis, and gluconeogenesis, which are commonly reported in diabetes. Based on our results, we may say that administration of ethanolic extract of *I. verum* decreased the ALP and SGPT levels in the plasma as compared to the diabetic group, and hence it may protect the liver tissues by decreasing the diabetes-induced oxidative stress. We may say that the liver-protecting effect of the *I. verum* dry fruit extract may be due to polyphenols and flavonoids contents (Table 3), exhibiting antioxidant effects.

Finally, we also evaluated the effect of *I. verum* dry fruit extract on renal AGE contents. STZ-induced diabetes caused a significant increase (*p* < 0.05) in the renal AGE content of the diabetic group as compared to the normal/ non-diabetic rats (Fig. 7). In the diabetic rats, renal damage was evident with an increase in serum urea, and creatinine levels (shown in Figs. 3, and 4), indicating reduced creatinine and urea clearance. Secondly, increased oxidative stress and impaired liver test parameters, *i.e.,* total bilirubin, direct bilirubin, indirect bilirubin, SGPT, Gamma GT, and ALP levels (Fig. 6) were also associated with an increased formation of renal AGEs in diabetic rats. AGEs are well known to be involved in structural changes in diabetic nephropathy, *e.g*., glomerulosclerosis, tubular atrophy, and interstitial fibrosis, hence affecting normal kidney functions. Blood urea, creatinine, and liver test parameters were reduced significantly on treatment with the dry fruit extract of *I. verum* (1,000 and 500 mg/kg of body weight). This effect was reflected in improved creatinine, blood urea, and renal AGEs levels in the diabetic rats treated with *I. verum* (1,000, and 500 mg/kg of body weight) extract, and consequently a decrease in non-enzymatic glycation. Based on these observations, it is established that the ethanolic dried fruit extract of *I. verum* had a positive influence against the onset of diabetes and AGEs-associated complications in streptozotocin-induced diabetic rats.

## Conclusion

Traditional plant-based therapies form an important part of diabetic treatments. We find out that dried fruits of a common spice, *Illicium verum* Hook. f. has the ability to reduce glycation, hyperglycemia, oxidative stress, and formation of AGEs (*in-vitro* and *in-vivo*), and thus may serve as an adjunct against diabetic, and non-enzymatic glycation reaction. Hence, it may be helpful in attenuating diabetic, and glycation associated complications. The evidences produced in this study are useful to design further pre-clinical, and clinical studies, and to investigate the anti-glycation agents from *I. verum* for the management of diabetic complications.

## Supplementary Information

**Additional file 1.**

## Data Availability

All data generated or analyzed during this study are included in this article.

## Abbreviations

AGEs: Advanced glycation endproducts
IDF: International Diabetes Federation
STZ: Streptozocin
DPPH: 1,1-Diphenyl-2-picrylhydrazyl
HSA: Human serum albumin
ABTS: 2,2′-azino-bis(3-ethylbenzothiazoline-6-sulfonic acid
EDTA: Ethylenediaminetetraacetic acid
TEMED: Tetramethylethylenediamine
SDS: Sodium dodecyl sulfate
SDS-PAGE: Sodium dodecyl sulfate polyacrylamide gel electrophoresis
DMSO: Dimethyl sulfoxide
RSA: Radical scavenging activity
Fe(II) chelation assay: Iron (II) chelation assay
SD rats: Sprague Dawley rats
SGPT: Serum glutamic pyruvic transaminase
ALP: Alkaline phosphatase
LFT: Liver function test
AU: Arbitrary units
HbA1c: Glycated haemoglobin
HDL: High-density lipoprotein
LDL: Low-density lipoprotein
VLDL: Very low density lipoprotein
Gamma-GT: Gamma-Glutamyl Transferase

## References

[1] International Diabetes Federation (2019). IDF Diabetes Atlas.

[2] Ahmed N (2005). Advanced glycation endproducts-role in pathology of diabetic complications. Diabetes Res Clin Pract.

[3] Yan JH, Xiao XX, Huang KL (2002). Component analysis of volatile oil from *Illicium Verum* Hook. f. J Cent South Univ.

[4] Wang GW, Hu WT, Huang BK, Qin LP (2011). *Illicium verum*: A review on its botany, traditional use, chemistry and pharmacology. J Ethnopharmacol.

[5] Patil SB, Ghadyale VA, Taklikar SS, Kulkarni CR, Arvindekar AU (2011). Insulin secretagogue, alpha-glucosidase and antioxidant activity of some selected spices in streptozotocin-induced diabetic rats. Plant Foods Hum Nutr.

[6] Chouksey D, Sharma P, Pawar RS (2010). Biological activities and chemical constituents of *Illicium verum* hook fruits (Chinese star anise). Der Pharmacia Sinica..

[7] Idm’hand E, Msanda F, Cherifi K (2020). Ethnopharmacological review of medicinal plants used to manage diabetes in Morocco. Clin Phytoscience.

[8] Shah H, Lohar M, Arora A, Kapoor C (2019). Study of ethno hypoglycemic food plants used by tribal’s of Southern Rajasthan (India). J Pharmacogn Phytochem.

[9] Choi SJ, Song YJ, Kim YS, Kim JH, Hang S, Kim JS (2012). Screening of herbal medicines from China and Vietnam with inhibitory activity on advanced glycation end products (AGEs) formation (VIII). Korean J Pharmacogn.

[10] Shahrajabian MH, Sun W, Cheng Q (2019). Chinese star anise and anise, magic herbs in traditional Chinese medicine and modern pharmaceutical science. Asian J Med Biol Res.

[11] Anise PE (2018). Antioxidant and anti-diabetic activities of polyphenol-enriched star anise (*Illicium verum*) seeds extract. Int J Biotechnol Biochem.

[12] Ahmad MS, Pischetsrieder M, Ahmed N (2007). Aged garlic extract and S-allyl cysteine prevent formation of advanced glycation endproducts. Eur J Pharmacol.

[13] Laemmli UK (1970). Cleavage of structural proteins during the assembly of the head of bacteriophage T4. Nature.

[14] Lee SK, Mbwambo ZH, Chung H, Luyengi L, Gamez EJ, Mehta RG, Kinghorn AD, Pezzuto JM (1998). Evaluation of the antioxidant potential of natural products. Comb Chem High Throughput Screen.

[15] Khatua S, Ghosh S, Acharya K. Simplified Methods for Microtiter Based Analysis of In Vitro Antioxidant Activity. Asian J Pharm. 2017;11(02):S327–5.

[16] Carter P (1971). Spectrophotometric determination of serum iron at the submicrogram level with a new reagent (ferrozine). Anal Biochem.

[17] Rasheed S, Sánchez SS, Yousuf S, Honoré SM, Choudhary MI (2018). Drug repurposing: *In-vitro* anti-glycation properties of 18 common drugs. PLoS One.

[18] Miliauskas G, Venskutonis PR, Van Beek TA (2004). Screening of radical scavenging activity of some medicinal and aromatic plant extracts. Food Chem.

[19] Brighente IM, Dias M, Verdi LG, Pizzolatti MG (2007). Antioxidant activity and total phenolic content of some Brazilian species. Pharm Biol.

[20] Zeb MA, Rahman TU, Sajid M, Xiao W, Musharraf SG, Bibi S, Akitsu T, Liaqat W (2021). GC-MS Analysis and In Silico Approaches of *Indigofera heterantha* Root Oil Chemical Constituents. Compounds.

[21] Sulaiman CT, Balachandran I (2016). LC/MS characterization of antioxidant flavonoids from *Tragia involucrata* L. Beni-Suef Univ J Basic Appl Sci.

[22] Percie du Sert N, Hurst V, Ahluwalia A, Alam S, Avey MT, Baker M, Browne WJ, Clark A, Cuthill IC, Dirnagl U, Emerson M (2020). The ARRIVE guidelines 2.0: Updated guidelines for reporting animal research. J Cerebral Blood Flow Metab.

[23] Hafizur RM, Babiker R, Yagi S, Chishti S, Kabir N, Choudhary MI (2012). The antidiabetic effect of Geigeria alata is mediated by enhanced insulin secretion, modulation of β-cell function, and improvement of antioxidant activity in streptozotocin-induced diabetic rats. J Endocrinol.

[24] Jagtap AG, Patil PB (2010). Antihyperglycemic activity and inhibition of advanced glycation end product formation by Cuminum cyminum in streptozotocin induced diabetic rats. Food Chem Toxicol.

[25] Elosta A, Slevin M, Rahman K, Ahmed N (2017). Aged garlic has more potent antiglycation and antioxidant properties compared to fresh garlic extract in vitro. Sci Rep.

[26] Li SN, Sun JF, Wang JM, Jin L, Zong TQ, Zhou W, Li G (2022). Two new phenolic glycosides from the fruits of *Illicium verum*. J Asian Nat Prod Res.

[27] Sharafan M, Jafernik K, Ekiert H, Kubica P, Kocjan R, Blicharska E, Szopa A (2022). *Illicium verum* (Star Anise) and Trans-Anethole as valuable raw materials for medicinal and cosmetic applications. Molecules.

[28] Sheikh BA, Pari L, Rathinam A, Chandramohan R (2015). Trans-anethole, a terpenoid ameliorates hyperglycemia by regulating key enzymes of carbohydrate metabolism in streptozotocin induced diabetic rats. Biochimie.

[29] Naksang P, Tongchitpakdee S, Thumanu K, Oruna-Concha MJ, Niranjan K, Rachtanapun C (2020). Assessment of antimicrobial activity, mode of action and volatile compounds of Etlingera pavieana essential oil. Molecules.

