# Supplementary material for: Anti-glycation properties of Illicium verum Hook. f. fruit in-vitro and in a diabetic rat model
Source: BMC Complement Med Ther. 2022 Mar 19;22:79. doi: 10.1186/s12906-022-03550-z (PMC8934496; doi:10.1186/s12906-022-03550-z)
Supplement: Supplementary file 1 — Additional file 1. [file 12906_2022_3550_MOESM1_ESM.docx]

**Supplementary Information**

**Anti-Glycation Properties of *Illicium verum* Hook. f. Fruit *In-vitro* and in a Diabetic Rat Model**


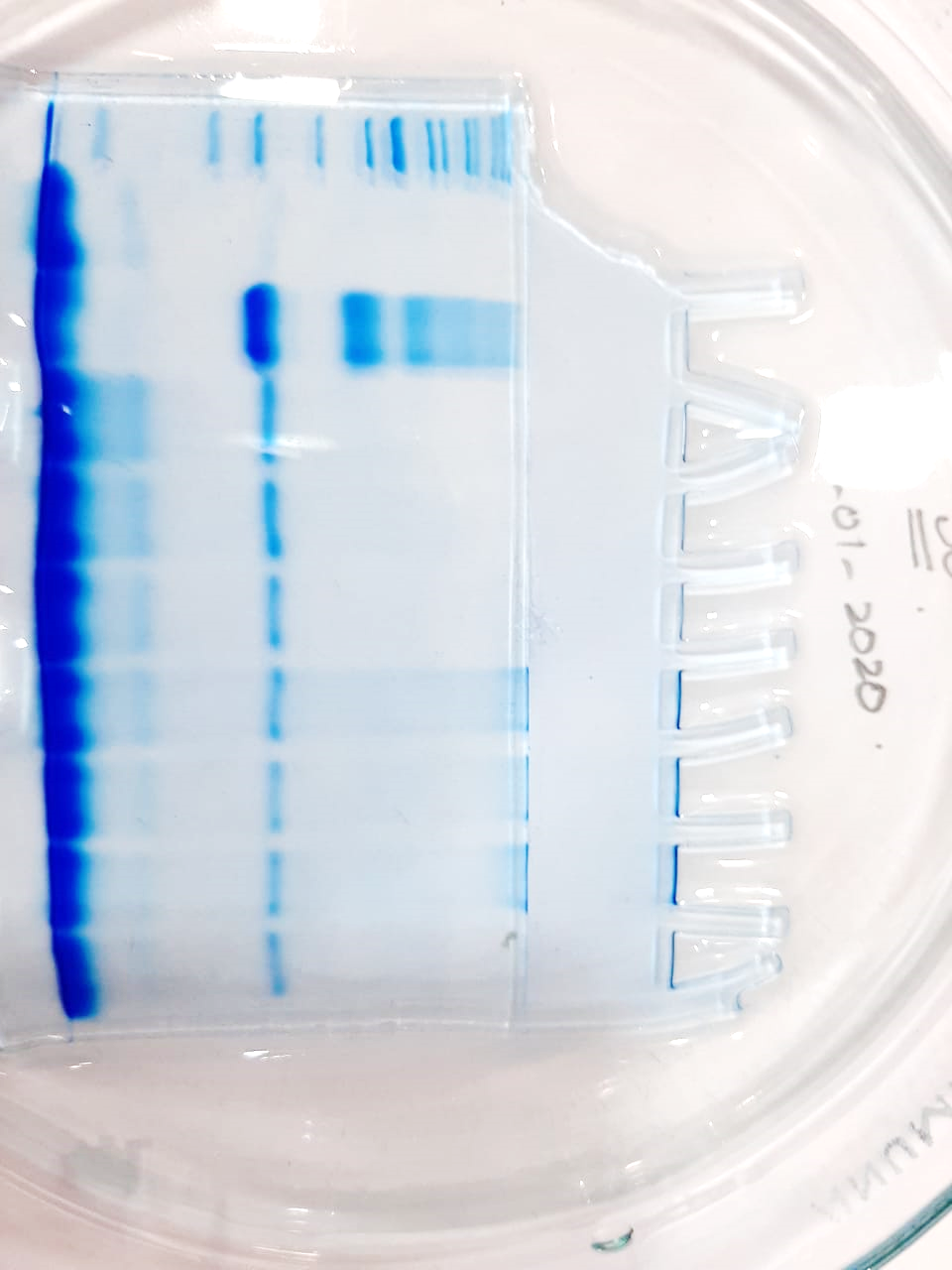


**Fig. S1.** Uncropped SDS polyacrylamide gel showing the anti-glycation activity of *Illicium verum* Hook. f. against glycated lysozyme (glycated in the presence of fructose).

**Fig. S2.** LC-MS base peak chromatogram (BPC) of the ethanolic extract of *I. verum.*

**Table S1.** Metabolites identified from ethanolic extract of *I. verum* after LC-MS analysis.

| **S. No.** | **Tentative identification** | **Molecular Formula** | **Exact Mass** | **Retention time (min)** |
| --- | --- | --- | --- | --- |
|  | 5,6-Dihydroxy-7-methoxyflavone | C_16_H_12_O_5_ | 284.068474 | 8.6 |
|  | Scutellarein | C_15_H_10_O_6_ | 286.047737 | 0.36 |
|  | 7-Hydroxy-3-(4-methoxyphenyl)-4-phenylcoumarin | C_22_H_16_O_4_ | 344.104858 | 6.16 |
|  | Jasmonic acid | C_12_H_18_O_3_ | 210.125594 | 9.12 |
|  | 3-Hydroxy-3',4'-dimethoxyflavone | C_17_H_14_O_5_ | 298.084124 | 13.71 |
|  | 3,4'-Dimethoxy-2-hydroxychalcone | C_17_H_16_O_4_ | 284.104858 | 15.9 |
|  | 9-Oxo-prosta-5*Z*,10,12*Z*,14*E*-tetraenoic acid | C_20_H_28_O_3_ | 316.203844 | 13.0 |

**Methodology:**

***In-vivo* studies:**

1. **Serum lipid levels**

Serum levels of lipid profile, including cholesterol, triglycerides, high density lipoproteins (HDL) and low density lipoproteins (LDL), liver functions test inclusive of bilirubin (total and direct) enzymes including Alanine aminotransferase (ALT), Alkaline phosphate (ALP) and cardiac markers including Aspartate aminotransferase (AST), Lactate dehydrogenase (LDH), Creatine kinase (CK), and Creatine kinase-MB (CKMB) were analyzed by fully automated Chemistry Analyzer, Hitachi 912 (Roche Diagnostics, Basel, Switzerland). For all these assays standardized kits were used by following provided methodology, calibrators and controls. Total lipid was estimated manually by calorimetric test method of phosphovanilline on Clinicon 4010 photometer (Boehringer Meannheim, Germany). Serum LDL-cholesterol concentration was also measured by using Friedwald formula where LDL-c= TC-(HDL-C)-TG/5 (Tsi *et al.,* 1995).

**1.1. Triglycerides:**

Serum triglycerides were estimated by GPO-PAP method by using kit which was acquired from Merck (Private) Limited, Pakistan. Serum (0.01 mL) sample was mixed with 1 mL of reagent solution and incubated for 5 min at 37 ºC. Absorbance was recorded at 500 nm [1].

**1.2. Cholesterol:**

Serum total cholesterol was measured by CHOD-PAP method by using kit which was acquired from Martin Dow Marker Specialties (Pvt.) Ltd. To determine cholesterol, 0.01 mL of sample was mixed with 1.0 mL of reagent solution. Sample and standard were incubated for 5 min. at 37 °C. Absorbance of samples were recorded against reagent at 500 nm [2].

**1.3. High-density Lipoprotein (HDL):**

Serum HDL was estimated by CHOD-PAP method by using kit which was acquired from Martin Dow Marker Specialties (Pvt.) Ltd. In 2.4 μL of sample, 240 μL of precipitant (reagent R1) were mixed and kept for 4 minutes and 40 sec. at 37 ºC, absorbance (A1) was then recorded at 578 nm. After that, 80 μL of cholesterol reagent (reagent R2) was added, mixed, and incubated for 4 minutes at 37 ºC. Absorbance (A2) was recorded at 578 nm [3].

**1.4. Low Density Lipoprotein (LDL):**

LDL-Cholesterol concentration was estimated by PVS method which was acquired from Merck (Private) Limited, Pakistan. Serum (2.4 μL) and precipitant (Regent R1; 240 µL) were mixed and kept for 4 minutes and 40 sec. at 37 ºC. Absorbance (A1) was then recorded at 578 nm. After that, 80 µL of cholesterol reagent (reagent R2; 80 μL) was added, mixed, and incubated for 4 minutes at 37 ºC. Absorbance (A2) was recorded at 578 nm [4].

**1.5. Very Low-Density Lipoprotein (VLDL):**

Serum VLDL was obtained by dividing TG with 5.

1. **Serum Glucose:**

GOP-PAP method (Trinder, 1969) of kit (which was acquired from Martin Dow Marker Specialties (Pvt.) Ltd.) was used to estimate the glucose levels in serum. 0.01 mL of serum was added in 1 mL of reagent solution and incubated for 5 minutes at 37 ºC. The absorbance of sample and standard were recorded against the blank at 500 nm [5].

1. **Estimation of Liver test parameters:**

**3.1. Total Bilirubin**

Total bilirubin was estimated by following kit (acquired from Martin Dow Marker Specialties (Pvt.) Ltd.) method. To determine the concentration of total bilirubin in serum samples, 30 µL of serum sample, and 240 μL of regent R1 were mixed and allowed to stand for 4.5 minutes at 37 °C. After incubation, absorbance (A1) was recorded at 546 nm. Then 80 µL of reagent R2 was added and incubated for 6.5 minutes at 37 °C. The absorbance (A2) was recorded at 546 nm against the sample blank [6].

**3.2. Direct Bilirubin**

Total bilirubin was estimated by following kit (acquired from Martin Dow Marker Specialties (Pvt.) Ltd.) method. To determine the concentration of total bilirubin in serum samples, 30 µL of serum sample, and 240 μL of regent R1 were mixed and allowed to stand for 4.5 minutes at 37 °C. After incubation, absorbance (A1) was recorded at 546 nm. Then 80 µL of reagent R2 was added and incubated for 6.5 minutes at 37 °C. The absorbance (A2) was recorded at 546 nm against the sample blank. The absorbance of the sample was read against the sample blank at 546 nm [6].

**3.3. Indirect Bilirubin:**

Serum indirect bilirubin was obtained by subtracting the value of direct bilirubin from total bilirubin.

**3.4. Alanine Aminotransferase (ALT):**

The serum ALT was assayed by the kit method, as recommended by IFCC (kit was purchased from Martin Dow Marker Specialties (Pvt.) Ltd.). 500 µL reagent solution was mixed with 50 µL of serum and absorbance was measured at 340 nm at 37 °C. Change in absorbance was recorded after one-minute interval for 3 minutes. To evaluate the value of ALT in IU/L, mean absorbance was multiplied with factor 2134 [7].

**3.5. Alkaline Phosphatase (ALP):**

The alkaline phosphatase was estimated by DGKC method by using kit (kit was purchased from Merck (Private) Limited, Pakistan). 10 µL of serum were mixed with 500 µL of reagent solution and absorbance was measured at 405 nm immediately (at 37 ºC). Change in absorbance was recorded after one-minute interval for 3 minutes. Changes in mean absorbance were multiplied with a factor of 2750 to get IU/L of ALP in serum sample [8].

**3.6. Aspartate Aminotransferase (AST):**

IFCC recommended kit method for AST estimation was used (kit was purchased from Martin Dow Marker Specialties (Pvt.) Ltd.). 50 µL of serum were mixed with 500 µL of reagent solution reagent solution were mixed, and after one minute 125 µL of start reagent solution was added, mixed and incubated for one minute. Absorbance was recorded every minute for 3 minutes at 340 nm. The same was repeated at every minute for three times. Mean change in absorbance was multiplied with a factor of 2143 to get IU/L of AST in serum [8].

**3.7. Gamma-glutamyl transpeptidase (****GGT):**

The serum GGT was assayed by the kit method, as recommended by IFCC (kit was purchased from Martin Dow Marker Specialties (Pvt.) Ltd.). 400 µL of reagent solution R1 and 100 µL of reagent solution R1 were mixed and incubated for 25 seconds, then 50 µL of serum sample(s) was mixed, and after 50 seconds of incubation, change in absorbance was measured per minute (ΔA/min.) during 3 minutes. To evaluate the value of GGT in U/L, ΔA/min. was multiplied with factor 1158 [8].

***References:***

1. Tietz, NW. and Finley, PR. Clinical guide to laboratory tests. 3^rd^ ed, Philadelphia: WB Saunders; 1995. 610.
2. Tietz, NW. and Finley, PR. Clinical guide to laboratory tests. 3^rd^ ed, Philadelphia: WB Saunders; 1995. 130.
3. Tietz, NW. and Finley, PR. Clinical guide to laboratory tests. 3^rd^ ed, Philadelphia: WB Saunders; 1995. 334.
4. Tietz, NW. and Finley, PR. Clinical guide to laboratory tests. 3^rd^ ed, Philadelphia: WB Saunders; 1995. 404.
5. Tietz, NW. and Finley, PR. Clinical guide to laboratory tests. 3^rd^ ed, Philadelphia: WB Saunders; 1995. 268.
6. Tietz, NW. and Finley, PR. Clinical guide to laboratory tests. 3^rd^ ed, Philadelphia: WB Saunders; 1995. 90.
7. Tietz, NW. and Finley, PR. Clinical guide to laboratory tests. 3^rd^ ed, Philadelphia: WB Saunders; 1995. 20.
8. Burtis CA, Ashwood ER, Border B., Tietz N W. Tietz fundamentals of clinical chemistry. 5^th^ ed. Philadelphia: W.B. Saunders; 2001. 352-87.
